# Regulatory T Lymphocytes Are Associated with Less Aggressive Histologic Features in Microsatellite-Unstable Colorectal Cancers

**DOI:** 10.1371/journal.pone.0061001

**Published:** 2013-04-15

**Authors:** David Tougeron, Pauline Maby, Nicolas Elie, Émilie Fauquembergue, Florence Le Pessot, Marie Cornic, Jean-Christophe Sabourin, Pierre Michel, Thierry Frébourg, Jean-Baptiste Latouche

**Affiliations:** 1 Inserm, U1079, Faculty of Medicine, Institute for Medical Research, Rouen University Hospital, Rouen, France; 2 Department of Gastroenterology, Rouen University Hospital, Rouen, France; 3 Department of Gastroenterology, Poitiers University Hospital, Poitiers, France; 4 Laboratoire Inflammation Tissus Epithéliaux et Cytokines, EA 4331, Poitiers University, Poitiers, France; 5 Plateau d’Histo-Imagerie Quantitative, CMABIO, Caen University, Caen, France; 6 Department of Pathology, Rouen University Hospital, Rouen, France; 7 Department of Pathology, Centre Henri Becquerel, Rouen, France; Yonsei University College of Medicine, Republic of Korea

## Abstract

**Background:**

Colorectal cancers (CRCs) with microsatellite instability (MSI) are associated with a good prognosis and a high density of tumor-infiltrating lymphocytes (TILs). We have undertaken to determine the link between TIL densities and MSI CRC histologic features.

**Patients and Methods:**

Using tissue microarrays, T-cell sub-population infiltration, including T cells (CD3), cytotoxic T cells (CD8) and regulatory T cells (FoxP3) were studied in 86 MSI CRCs. We separately analyzed TILs of the stromal and epithelial compartments in the tumor center, the tumoral invasion margin and associated normal tissue.

**Results:**

For FoxP3+ TIL density in the tumor center stromal compartment, we found a strong negative correlation with T4 stage (p = 0.01), node invasion (p<0.001) and VELIPI (vascular emboli, lymphatic invasion and perinervous invasion) criteria (p = 0.002).

**Conclusion:**

The strong correlation between regulatory T cell density and the absence of VELIPI criteria suggests that this sub-group of T cells is preferentially associated with less invasive tumors.

## Introduction

Recently, host immune response has been shown to play an important role in determining the outcome of patients with colorectal cancer (CRC). Pages *et al* have demonstrated that a high density of infiltrating memory T cells within the tumor is associated with the absence of histological markers of ongoing invasion, also called VELIPI (vascular emboli, lymphatic invasion and perinervous invasion) criteria and with better overall survival [1], [2]. Moreover, tumor-infiltrating lymphocyte (TIL) density seems to have a better prognosis value than TNM stage [3], and some T cell subpopulations have been shown to be associated with a better CRC clinical outcome, such as cytotoxic T cells (CD8+), Th17 helper T cells and, more controversially, regulatory T cells (FoxP3+) [3]–[5].

Interestingly, tumors presenting a microsatellite instability (MSI) or replication error phenotype are histopathologically characterized by high TIL density [6]–[8]. These tumors have an overall better prognosis with less disease recurrence and better survival rate than the other CRC counterpart, with microsatellite stable phenotype [9], [10]. MSI phenotype, observed in approximately 12% of colorectal cancers, is due to the inactivation of the DNA mismatch repair (MMR) system^11^ that results either from a somatic epigenetic alteration, or from a germline alteration (Lynch syndrome) [11]. MMR inactivation affects nucleotide repeat replication and can lead to the generation of neo-proteins degraded in neo-peptides presented on HLA molecules. Therefore, the better prognosis for microsatellite unstable colorectal cancers could be at least partially explained by the presence of TILs which could be specific to certain tumor peptides resulting from microsatellite instability [12], [13]. MSI tumors have higher rates of CD8+ and FoxP3+ TILs than MSS tumors but the few published studies on MSI CRCs report contradictory results regarding these TIL prognosis values [4], [8], [14]–[18]. Moreover, in MSI CRCs, only CD8+ intraepithelial lymphocyte density has been fully studied, excluding stromal lymphocytes and other T cell sub-populations [14], [19].

Immune cell density might have a prognostic value, and therefore be clinically relevant, especially for prescription of adjuvant chemotherapy in MSI CRCs. But to our knowledge, up until now no study has investigated, in a large series of patients, the link between the different TIL sub-populations in the stromal and epithelial compartments, and prognosis or tumor histologic features, in MSI CRCs. In this study, we have analyzed total T cell, cytotoxic T cell and regulatory T cell tumor infiltration in MSI CRCs and tried to establish this link for these immune cell populations.

## Materials and Methods

### Patient Selection and Microsatellite Instability Assessment

In our institution, microsatellite instability has been analyzed in all CRCs since 2003. For each patient, genomic DNA was extracted from paired tumor and normal colorectal tissues. Microsatellite instability assessment was performed by a comparative analysis of these DNA samples using the five consensus mononucleotide repeats (*BAT25*, *BAT26*, *NR21*, *NR22* and *NR24*) [20]. Microsatellite instability (MSI) was defined by the presence of instability affecting at least two markers (corresponding in fact to MSI high tumors only). Our institution’s Ethics Committee (Comité de protection des personnes Nord-Ouest I) approved the study and waived the need for informed consent because of the retrospective nature of the study. In accordance with French regulations, written consent had systematically been obtained for the DNA mismatch repair system germline mutation analyses which were performed for medical purposes.

### Patient and Tumor Characteristics, and Clinical Outcome

Patient and tumor baseline characteristics were collected. Moreover, for each tumor, histological markers of ongoing invasion corresponding to the VELIPI criteria were determined by a pathologist’s examination [1].

All patients less than sixty years old and/or with familial history of Lynch syndrome tumor spectrum were addressed to oncogenetic consultation. In these patients, the presence of a deleterious germline DNA mismatch repair system gene mutation was sought out.

Disease recurrence was evaluated every 3 months the first two years and every six months the next three years. Follow-up data were updated in December 2011.

### Tissue Microarrays and Immunohistochemistry

Tissue microarrays (TMA) consist of paraffin blocks in which up to 1000 separate tissue cores are assembled in an array fashion to allow for multiple histological analyses after immunostaining. For each tumor, four cores of 0.6 mm diameter were taken at the tumor center, at the invasion margin, and four additional cores were taken from distant histologically normal colonic mucosa (Tissue Arrayer, Alphelys, France). All analyses were performed on surgicaly resected primary CRC before chemotherapy treatment. Area with Crohn’s like lymphoid response were excluded from TMA construction and analyses. The eight TMAs used in this study contained more than 1000 cores of microsatellite unstable CRCs. Four µm-thick sections were cut from the TMA blocks and used for immunohistochemistry based on CD3 (T cell), CD8 (cytotoxic T cell) and FoxP3 (regulatory T cell) staining. Formalin-fixed tissues were deparaffinized and heat-induced epitope retrieval was performed using a steamer. Slides were incubated with mouse monoclonal antibodies, anti-CD3 (DAKO, Carpentria, CA) diluted 1/50 for 32 min, anti-CD8 (DAKO, Carpentria, CA) diluted 1/50 for 32 min, and anti-FoxP3 (ABCAM, Cambridge, MA) diluted 1/50 for 32 min, according to the manufacturers’ instructions. For each section, a mouse primary antibody and a secondary anti-mouse antibody for revelation were used. The secondary antibody was directly coupled to HRPO enzyme (Horse-radish-peroxydase, DAKO) and applied for 15 min. Color was revealed using DAB chromogen (Diaminobenzine) and H2O2 (hydrogen peroxide) application.

### Quantification of Infiltrating Lymphocytes

**Figure 1:**
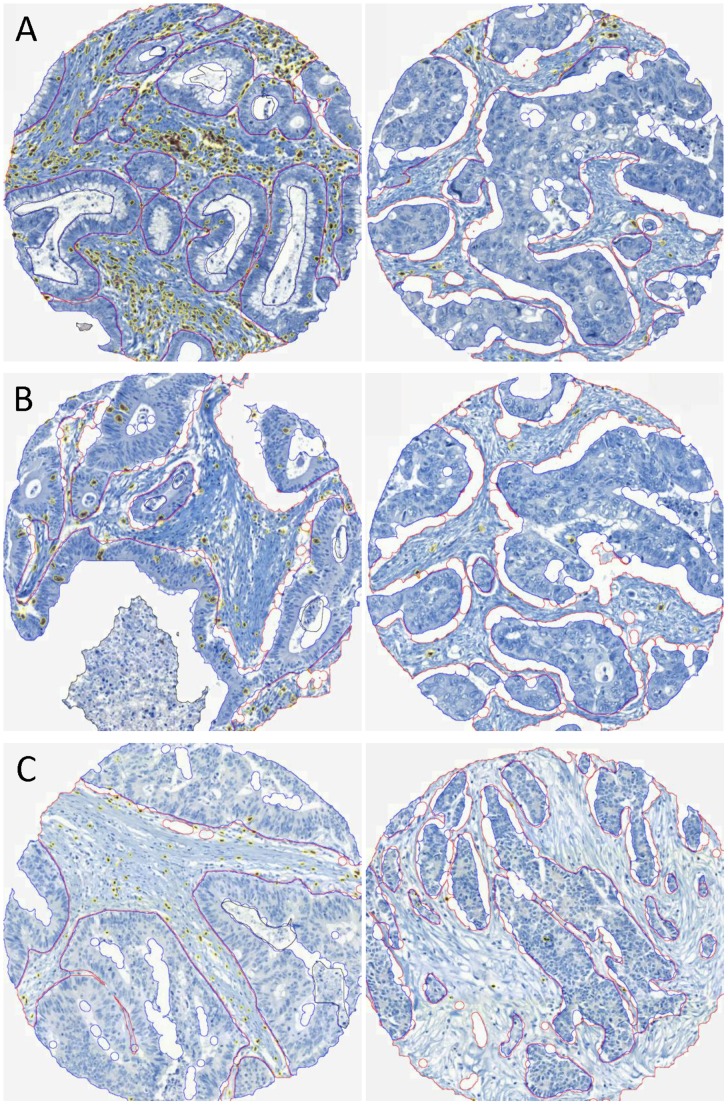
Determination of tumor-infiltrating lymphocyte density. Examples of tumor center chips with CD3 (A), CD8 (B) and FoxP3 (C) staining. Left panels are examples of high TIL densities and right panels are examples of low TIL densities. The stroma is circled in red, the epithelium in blue, and black areas are excluded. The marked areas that are taken into account to calculate the lymphocyte infiltrate density are circled in yellow. All data were acquired with Chip’s N Cheap TMA analysis program.

Each slide was scanned with Mirax Scan®. The Chips’N Cheap TMA Analysis program, using Aphelion 3.2 (ADCIS, Hérouville St Clair, France), had been optimized to identify each chip on the TMA virtual slide and to allow us to delineate the acellular areas, the stromal and the epithelial compartments for each core. The regions of interest, i.e. stroma and epithelium, were manually delineated within the non-malignant (4 cores), tumor center (4 cores), and invasion margin (4 cores) sites for each patient. After substraction of the acellular areas, the ratios between immunostained areas and total tissue region areas were calculated automatically (Figure 1).

TIL quantifications were given by the percentages of the total surfaces labeled by the antibodies. Visual analysis of the samples from 10 patients led us to estimate that one percent of tumor- infiltrating lymphocyte staining corresponded to around 350 TILs/mm^2^. In tumors with mucinous differentiation, TIL densities were calculated outside of the mucinous areas.

### Statistical Analysis

T-test was used to test association between tumor characteristics and TIL density. Disease-free survival (DFS) was estimated starting from the surgery date until the documented recurrence or last follow-up date. Survival curve was established using the Kaplan–Meier method. All statistical analyses were performed with a two-side significance value of 0.05, using the Statview software (Statview for Windows, SAS Institut Inc., version 5.0).

## Results

### Patient and Tumor Characteristics

A total of 106 CRCs with microsatellite instability (two patients developed 2 synchronous tumors), who had undergone surgery between January 2003 and December 2009, were initially included. Some patients were subsequently excluded from the analysis due to absence of tumor sample or clinical data. Eighty-six patients were still included in the final study (Table 1). Mean patient age was 68.6±17.5 years. Thirty-one patients were tested for MMR gene germline mutations. Eighteen of the patients (24.4%) presented a Lynch syndrome, as defined by the presence of a deleterious germline MMR mutation (16 patients) or a presentation fulfilling Amsterdam criteria in patients without germline MMR mutation (2 patients).

**Table 1:** Table **1.** Patient and tumors characteristics.

n = 86		
**Age (years)**		68.6±17.5
**Men/Women**		39/47
**Tumor location**		
	rectum	6 (7.0%)
	left colon	22 (25.6%)
	right colon	55 (63.9%)
	unknown	3 (3.5%)
**Wall invasion**		
	T1	3 (3.5%)
	T2	2 (2.3%)
	T3	47 (54.6%)
	T4	29 (33.7%)
	unknown	5 (5.8%)
**Node invasion**		
	N0	44 (51.1%)
	N+	35 (40.7%)
	unknown	7 (8.1%)
**TNM stage**		
	I	5 (5.8%)
	II	39 (45.3%)
	III	25 (29.0%)
	IV	10 (11.6%)
	unknown	7 (8.1%)
**Tumoral differentiation**		
	well	42 (48.8%)
	moderate	22 (25.6%)
	poor	15 (17.4%)
	unknown	7 (8.1%)
**VELIPI**		
	no	35 (40.7%)
	yes	39 (45.3%)
	unknown	12 (13.9%)
**MMR mutation**		16 (18.6%)
**Adjuvant chemotherapy**		21 (24.4%)
**Disease recurrence**		16 (18.6%)
**Death**		37 (43.0%)
	due to cancer	31 (36.0%)
	other causes	6 (7.0%)
**DFS (months)**		not reached
**Overall survival (months)**		72.1±11.3

n: number of patients, VELIPI: vascular emboli or lymphatic invasion or perineural invasion.

A majority of the tumors were TNM stage II or III (74.4%) and in the right colon (63.9%) (Table 1). Around half of the tumors had VELIPI criteria (45.3%) and 12.7% were mucinous tumors, defined as tumors with mucinous differentiation representing at least 50% of the total tumor volume; 25.3% of all the tumors had some mucinous differentiation representing less than 50% of the total tumor volume (less than 20% for almost half of these tumors, and between 20 and 40% for the others). Only 16 (18.6%) patients relapsed after a median follow-up of 25.0±5.7 months. Then, median DFS was not reached. Three-year DFS was 78.7%. Median overall survival (OS) was 72.1±11.3 months and three-year OS was 60.2%.

### Tumor-infiltrating Lymphocytes

**Figure 2:**
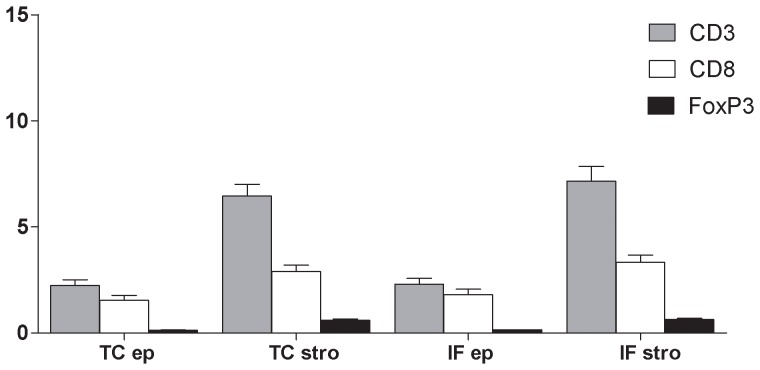
Percentages of tumor-infiltrating lymphocytes in the different areas studied. The bar charts represent the percentages of surfaces marked by CD3, CD8 and FoxP3 immunostaining in the stromal (stro) and epithelial (ep) compartments, in the tumor center (TC) and the invasion front (IF) tissues. Standard errors are given by the bars. CD3+ TIL densities were respectively, 2.2±2.5% in tumor center epithelium compartment, 6.4±5.1% in tumor center stromal compartment, 2.3±2.5% in invasion front epithelium compartment, 7.2±6.1% in invasion front stromal compartment. CD8+ TIL densities were respectively, 1.52±2.0% in tumor center epithelium compartment, 2.9±2.7% in tumor center stromal compartment, 1.8±2.4% in invasion front epithelium compartment, 3.3±3.0% in invasion front stromal compartment. FoxP3+ TIL densities were respectively, 0.13±0.12% in tumor center epithelium compartment, 0.59±0.53% in tumor center stromal compartment, 0.14±0.14% in invasion front epithelium compartment, 0.63±0.58% in invasion front stromal compartment.

CD3+, CD8+, and FoxP3+ T cells were predominantly localized in the stromal compartment (Figure 2). A significantly higher total T cell density was observed in tumor as compared to normal colon tissues (p<0.05, data not shown), as already reported.^13^ Within the tumor and normal tissues, CD3+, CD8+ and FoxP3+ TIL densities were higher in the stroma than in the epithelium (p<0.01 for all markers) (Figure 2). In addition, there was a strong positive correlation between CD3+, CD8+ and FoxP3+ TIL densities observed in both the tumor center and the invasion front, regardless of the compartment (p<0.01, data not shown). Moroever, there were higher CD8+ TIL densitites in the invasive front than in tumor center (p<0.05), which was not the case for CD3+ and FoxP3+ TIL densities. There was no difference between TIL densitites in patients with or without Lynch syndrome.

### Association between TIL Densities and Clinicopathological Variables

We looked for a correlation between TIL densities in the different compartments and a number of different clinicopathological variables (> 60 years versus ≤ 60 years, poorly versus well/moderately differentiated tumors, rectal/left colon versus right colon tumor localization, wall invasion (T4 versus T1–3), node invasion (N+ versus N0), histological markers of ongoing invasion (VELIPI criteria), stages I-II versus III-IV). CD3+ TIL density in the tumor center epithelium compartment was associated with poor differentiation (p = 0.01, data not shown). For CD3+ TIL density in the other compartments and CD8+ TIL density in all compartments, there was no correlation with clinical data. On the contrary, FoxP3+ TIL density in the tumor center stromal compartment was strongly negatively associated with T4 stage (p = 0.01), node invasion (p<0.001), VELIPI criteria (p = 0.002) and stages III-IV (p<0.001) (Table 2). There was no correlation between FoxP3+ TIL density in any other compartment and clinical data.

**Table 2:** Correlation between FoxP3+ tumor-infiltrating lymphocyte (TIL) density in stromal compartment of tumor center and clinicopathological variables.

		% of FoxP3+ TILs in stromal tumor center	p
**Age**			0.76
	>60 years	0.58±0.27	
	≤60 years	0.62±0.33	
**Tumor differentiation**			0.36
	well/moderate	0.61±0.28	
	poor	0.48±0.14	
**Tumor location**			0.21
	rectal/left colon	0.47±0.19	
	right colon	0.62±0.26	
**Wall invasion**			0.01
	T1–3	0.69±0.31	
	T4	0.40±0.08	
**Node invasion**			<0.001
	N0	0.76±0.30	
	N+	0.34±0.06	
**TNM stage**			0.001
	I–II	0.78±0.31	
	III–IV	0.36±0.07	
**VELIPI**			0.002
	no	0.84±0.34	
	yes	0.41±0.08	

TILs: tumor-infiltrating lymphocytes, VELIPI: vascular emboli or lymphatic invasion or perineural invasion.

## Discussion

In this study we initially undertook to evaluate the prognostic values of different TIL (CD3+, CD8+, FoxP3+) densities in colorectal cancers with microsatellite instability. In univariate analysis, node invasion (p = 0.002) and VELIPI criteria (p = 0.004) were predictive of DFS, but not CD3+, CD8+ and FoxP3+ TIL densities (data not shown). Although our large series of patients (n = 106) was reliable to study different histologic features of CRCs with microsatellite instability, it was not sufficient to carry out a robust statistical analysis concerning survival. Indeed, because this type of cancer has a good prognosis, only a few patients (n = 16) had relapsed and no correlation between any TIL population density and DFS could be observed. Nevertheless, we found a strong negative correlation between FoxP3+ TIL density in the tumor center stromal compartment and more aggressive tumor features: wall invasion, node invasion and VELIPI criteria, suggesting that the regulatory T cells within the tumor could be associated with limited cancer cell propagation potential.

Several studies have shown that CD8+ T cells, which appear to constitute the majority of the intra-epithelial infiltrate, are associated with better survival in CRCs [21]–[24]. Moreover, the overall CD8+ TIL infiltrate was shown to be more dense in MSI CRCs than in MSS CRCs, probably due to the expression by MSI tumor cells of frameshift mutation-derived immunogenic neoantigens [13]–[17]. These studies suggest that the survival advantage in MSI CRCs might be, at least in part, attributed to increased CD8+ intra-tumor T cell response, targeted against these neoantigens. However, our study did not establish association between CD8+ infiltrate density and better prognosis in MSI CRCs. It would be interesting to evaluate the functionality of the CD8+ TILs since some clinical observations suggest an impaired function of the CD8+ TILs in MSI CRCs [25], [26].

Another important TIL subpopulation is composed of CD4+ T cells. Unfortunately, up until now, we have not been able to analyze the CD4+ T cell population, even though effective CD8+ cytotoxic responses require the help of CD4+ T cells. Indeed, among the different anti-CD4 antibodies we tried out, not a single one allowed for staining of good quality that would be usable with regard to TMA and compatible with our image analysis software. CD4+ T cells appear increased in tumors in comparison with normal tissues [27] and a low CD4+ infiltrate may be associated with better survival [28], [29], which remains controversial [5]. This observation could be explained by the fact that CD4+ T cells represent a heterogeneous population containing both activated helper T cells of diverse activities, and regulatory T cells.

Great interest has recently been shown in FoxP3+ T cells that have regulatory functions on the immune response. This lymphocyte population is characterized by secretion of immunosuppressive cytokines (TGF-ß and IL-10) and expression of molecules such as CTLA-4 (Cytotoxic T-Lymphocyte Antigen 4) that inhibit activation and proliferation of CD4+ and CD8+ effector T cells [30]. A high density of regulatory T cells has been found frequently in the lymph nodes and tumors of CRC patients, and also in their blood, compared to healthy individuals [31]–[33]. Initially, some studies found a correlation between high FoxP3+ TIL density and poor prognosis in CRC [4], [34]. Recent studies, however, have surprisingly found that this infiltrate might be associated with a good prognosis [33], [35]–[37]. To date, only Michel *et al* have thoroughly evaluated the presence of these cells in MSI CRCs [38]. They observed a higher FoxP3+ infiltrate in MSI CRCs than in MSS CRCs. However, the link with prognosis was not studied. In our study, in MSI CRCs, FoxP3+ TIL density was clearly associated with less invasive tumor features, in particular with the absence of VELIPI criteria, and therefore could be indirectly associated with a better prognosis. This result was unexpected and may even seem paradoxical since regulatory T cells inhibit immune response. In fact, these regulatory T cells could reflect a mechanism developed by the tumor itself to limit anti-tumor response, and, perhaps, the more efficient this anti-tumor response is, the more numerous these regulatory T cells are. This hypothesis is strongly supported by the correlation we found between CD3, CD8 and FoxP3+ TIL densities. Another explanation coul be that regulatory T cells reduce inflammation which can contribute to tumor growth and its metastatic potential, as reported by Mantovani *et al* [39]. Heterogeneity in colorectal cancer infiltrating regulatory T cells, as recently described by Blatner *et al* [40], could also explain some unexpected clinical outcomes in colorectal cancer patients. However, no statistically significant correlation between FoxP3+ TIL density and DFS could be established, perhaps again because of a lack of statistical power due to the small number of tumor recurrences.

A strength of our study compared to other studies was that we took into account the precise TIL localizations. The different TIL subpopulations appeared to be mainly co-localized in the tumor stroma. As in MSS CRCs, CD8+ T cells appeared to constitute the majority of the intra-epithelial T lymphocyte infiltrate in MSI CRCs. FoxP3+ T cell density appeared to be low as compared to CD3+ and CD8+ T cells, highlighting the fact that other CD4+ T cells are present, which could play an important role in MSI CRCs.

In our study, predictive factors of recurrence were node invasion and VELIPI criteria, as already reported in MSS CRCs [41]. Association between tumor-infiltrating regulatory T cell density and the absence of VELIPI criteria is certainly the most important finding in this study. All these results need to be confirmed and more precisely defined, hopefully in a larger multicentric cohort of MSI CRC patients, including more patients with disease recurrence.
